# The most virulent parasite determines virulence in coinfection: a meta-analysis

**DOI:** 10.1017/S003118202610170X

**Published:** 2026-04

**Authors:** Charlotte Rafaluk, Victoria L. Pike, Mathias Franz, Kayla C. King

**Affiliations:** 1 Evolutionary Biology, Freie Universität Berlin, Berlin, Germany; 2 Department of Biology, University of Oxford, Oxford, UK; 3 Department of Zoology, University of British Columbia, Vancouver, Canada; 4 Department of Microbiology & Immunology, University of British Columbia, Vancouver, Canada

**Keywords:** coinfection, competition, host–parasite interaction, transmission, virulence

## Abstract

Coinfections of hosts by multiple parasite species and strains are widespread in nature. Theory suggests that these infections have a key influence on the virulence, or harm caused, to hosts. However, it is still unclear whether multiple parasites, which may compete for resources and space, are indeed worse for hosts across the tree of life. To test this hypothesis, we conducted separate meta-analyses based on different expectations derived from virulence in single infections. We included 68 effect sizes from 19 experiments on non-human animal host species and 38 parasite species combinations. We found that coinfections are overall more virulent than the mean degree of harm caused by both parasites in retrospective single infections. That said, the coinfection virulence level is similar to that of the most virulent parasite, and less than the additive virulence of both single infections. These results suggest that the most virulent parasite is the primary driver of virulence in coinfection. This finding has implications for parasite spread in nature and suggests we focus on controlling the more harmful parasites in the first instance, when trying to limit the damage caused by coinfection.

## Graphical abstract

**Figure:**
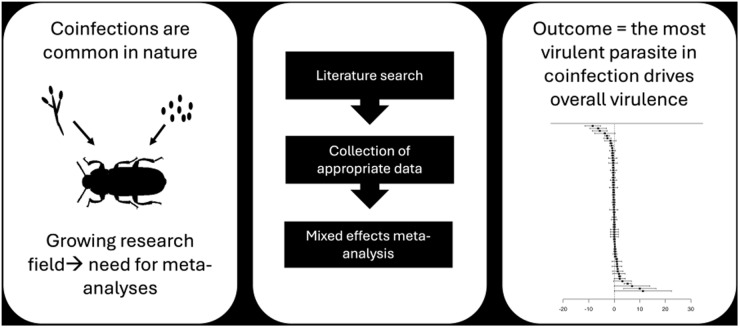

## Introduction

A parasite rarely infects a host alone (Petney and Andrews, 1998; Cox, 2001; Pedersen and Fenton, 2007; Rynkiewicz et al., 2015; Betts et al., 2016b; Hoarau et al., 2020). Most hosts are simultaneously infected by several parasite genotypes or species in coinfections (Cox, 2001). Coinfections are highly prevalent in humans (Petney and Andrews, 1998; Griffiths et al., 2011, 2015). For example, coinfections of human influenza virus with tuberculosis (Lawn et al., 2006; Harries et al., 2010; Kwan and Ernst, 2011) and malaria (Abu-Raddad et al., 2006) are common. Diverse communities can be found infecting hosts across the tree of life, from ticks (Moutailler et al., 2016) and voles (Telfer et al., 2010) to bacterial hosts (Betts et al., 2016a; Díaz-Muñoz, 2017) and to plants (Tollenaere et al., 2016). A similar diversity is found amongst the coinfecting parasites. Coinfections can occur between phylogenetically divergent species, such as helminth and malaria infections (Brooker et al., 2007; Mazigo et al., 2010; Knowles, 2011), to more similar parasites (Lord et al., 1999), such as a mixture of influenza A genotypes (Sharp et al., 1997). Despite the ecological (Tompkins et al., 2011), biomedical (Griffiths et al., 2011; Vaumourin et al., 2015) and agricultural (Figueroa et al., 2017; Liu et al., 2020) importance of coinfections, historically, studies have largely focused on single infections (Pedersen and Fenton, 2007; Rigaud et al., 2010; Telfer et al., 2010; Eswarappa et al., 2012). However, over the past 10–15 years, interest in coinfection has begun to increase (Bose et al., 2016; Tollenaere et al., 2016).

Coinfecting parasites can interact within hosts (Read and Taylor, 2001; Pedersen and Fenton, 2007; Buckling and Brockhurst, 2008; Rynkiewicz et al., 2015), and the mechanism of interaction is predicted to drive changes in host harm or virulence over evolutionary time (May and Nowak, 1995; Frank, 1996; Alizon et al., 2013). The direction in and extent to which virulence changes in coinfections compared to single infections is not necessarily additive (Rigaud et al., 2010; Bordes and Morand, 2011; Eswarappa et al., 2012; Alizon et al., 2013; Vaumourin et al., 2015). Coinfecting parasites may have overlapping ecological niches encouraging within-host resource competition (Read and Taylor, 2001; Mideo, 2009), such as when the protozoan *Babesia microti* and the bacterium *Bartonella* spp. compete for red blood cells within their field vole (*Miavtus agrestis*) host (Telfer et al., 2010). Virulence may similarly be increased by parasite facilitation in coinfections. For example, within mice Plasmodium-induced lysis of red blood cells causes iron to be released which in turn increases *Salmonella* growth, leading to an increased overall virulence (Kaye and Hook, 1963; Kaye et al., 1965; Uneke, 2008; Eswarappa et al., 2012). Not all coinfection leads to increased virulence, however. There are examples across systems of both antagonistic and facilitative interactions between coinfecting parasite species (Syller, 2012; Manna et al., 2022).

While theory tends to address the extent to which virulence can evolve during coinfections, empirical research has tended to test effects of coinfection on individual hosts over ecological time scales. Understanding the generality of the impact of coinfections on individual hosts is crucial for predicting disease outcomes and the development of effective treatments. This understanding is particularly important when novel coinfections arise (Hassell et al., 2021), such as between COVID-19 and influenza A virus (Bai et al., 2021). Furthermore, the presence of coinfecting parasites can complicate treatments (Pedersen and Antonovics, 2013; Ezenwa and Jolles, 2015; Vaumourin et al., 2015; Clerc et al., 2019; Ezenwa et al., 2021) and few guidelines exist for the treatment of specific coinfections within humans (Griffiths et al., 2015). Thus, quantitatively assessing broad patterns may be applicable for treatment approaches potentially based on parasite–parasite interactions (Griffiths et al., 2015) across clinical and ecological settings.

We addressed the generality of the impact of coinfections on virulence with a formal meta-analysis. We searched the published literature for all available data sources focusing on experimental studies in animal hosts, to rigorously assess the relationship between coinfection and virulence. We chose animal hosts as we chose to focus on mortality-based measures of virulence in order to be able to compare coinfections to different baseline measures of virulence in single infection and because there are very few studies measuring mortality virulence in plants. We use the term ‘parasite’ throughout to refer to micro- and macro-parasites, as it is frequently used in ecological and evolutionary studies (Tompkins et al., 2011; Eswarappa et al., 2012; Alizon et al., 2013). We estimated the effect of coinfection on parasite virulence using Glass’s Δ as a measure of effect size. We explored whether the virulence effect size was moderated by the phylogenetic distance between coinfecting parasites, different transmission modes (i.e. whether the parasite was transmitted via host–host contact – including vertical transmission – or indirectly, for example via vectors, water or the environment), and whether the parasites shared a host site of infection or not.

## Materials and methods

### Literature search

We conducted a literature search on papers published up to and including 23rd March 2023 on Web of Science (see Figure S1 for PRISMA flow diagram (Moher et al., 2009; Page et al., 2021)). We recorded the number of studies published per year with the term ‘coinfection’ or ‘co-infection’ as we wanted to track any trends in publishing over time and we also collected this data for the term ‘virulence’ for comparison. We performed forwards (looking through the references citing each included paper) and backwards (looking through the references cited by each included paper) citation searches on the papers of interest. Papers were included in this study if they fit the following criteria:

1. The studies measured parasite virulence (harm caused to the host) *in vivo*.
2. The study collected a mortality-based measure of parasite virulence from both parasite genotypes or species in single infection (virulence of parasite A and virulence of parasite B).
3. The study collected an overall measure of parasite virulence in coinfection (virulence of coinfection).
4. Virulence must be measured as time to death, proportion or percentage alive or LT_50_ in order to calculate the different expectations for coinfection.
5. In the cases of proportions and percentages, the virulence measure used must be >0% and <100% for both parasites in single infection.
6. The study tested no more than 2 parasite genotypes or species in coinfection simultaneously.

Criteria 2 and 3 enabled us to directly compare the virulence of each parasite in single infection and coinfection and led to the exclusion of all studies on humans and most in mice. This criterion was particularly important as the direct comparison allowed us to determine that changes in virulence were the result of the addition of a coinfecting parasite, rather than the fact that the parasite chosen for comparison was the less virulent of the 2, and also to determine the kind of interaction between the parasites in coinfection, i.e. whether virulence is additive or not. Furthermore, to meet these strict criteria, all the studies in our analysis were experimental.

We define virulence as the degree of harm caused to the host from infection. We recorded the type of measure of virulence to compare differences between the types of measurement. We only used virulence measures that used survival data. We also collected data on parasite taxonomic grouping (at all levels from kingdom to species), transmission mode (direct i.e. host-to-host or indirect i.e. via vector, environment or water), site of infection (the same or different to that of the coinfecting parasite) and host species. In total, we found 33 papers (see Table S1) that fulfilled all our inclusion criteria which incorporated a range of hosts (birds, fish, insects, mammals, nematodes and zooplankton) and parasites (bacteria, fungi, viruses, nematodes, microsporidia, protozoa and trematodes).

### Meta-analysis

We carried out a series of multivariant meta-analyses using the rma.mv function in the ‘metafor’ package in R, following the methods of Becker et al. (2015) and Gibson and Nguyen (2021). As multiple effects were extracted from some studies and certain host and parasite species were used in multiple studies, ‘study’, ‘parasite one species’, ‘parasite two species’ and ‘host species’ were included as random effects in the model. Moderator variables were sequentially included as fixed effects. A full list of moderators tested can be found in Table 2.

We carried out 3 meta-analyses in order to assess how the virulence of single infections is related to the virulence in the corresponding coinfection. Specifically, we considered 3 different ways of how single infection virulence can be used to calculate the expected coinfection virulence (Table 1). For each expectation, which differed in the level of expected virulence, we carried out a meta-analysis to assess whether the observed coinfection virulence systematically differed from this expectation. For our low virulence expectation, we calculated the mean virulence of both single infections. Our medium virulence expectation was based on the highest virulence of both single infections. Finally, as a high virulence expectation, we calculated the additive virulence of both single infections.

**Table 1.** Calculation of expectations of mean and additive virulence for different virulence measures. Measures of survival in single infections are denoted *s*_1_ and *s*_2_. To derive our expectations, each virulence measure was first transformed into the underlying host survival rate assuming a constant rate throughout the experiment. After calculating mean and additive rates, the expectations were then back-transformed again into the respective measure. The equations reflect the outcome of these different steps Table 1 long description.

	Proportion survived	Percent survived	Mean survival time	LT_50_
Mean virulence	$\sqrt{s_{1}s_{2}}$	$\sqrt{s_{1}s_{2}}$	$\frac{1}{\frac{1}{2s_{1}}+\frac{1}{2s_{2}}}$	$\frac{\text{log}\left(2\right)}{\frac{\text{log}\left(2\right)}{2s_{1}}+\frac{\text{log}\left(2\right)}{2s_{2}}}$
Additive virulence	$s_{1}s_{2}$	$\frac{s_{1}s_{2}}{100}$	$\frac{1}{\frac{1}{s_{1}}+\frac{1}{s_{2}}}$	$\frac{\text{log}\left(2\right)}{\frac{\text{log}\left(2\right)}{s_{1}}+\frac{\text{log}\left(2\right)}{s_{2}}}$

We aimed to avoid biases in our expectations caused by the specific virulence measured used. The basic idea of the approach we took was to derive mean and additive expectations for all 4 virulence measures based on the underlying death rate. These expectations were then again back transformed into the corresponding virulence measure to allow a comparison to the measured coinfection virulence. For this reason, we only included studies with mortality-based measures of virulence. We assumed that for any virulence measure, the underlying virulence is reflected by the average survival rate during the experiment, and our expectations were therefore based on mean and additive survival rates. Table 1 lists the equations of the corresponding calculations, which include the back-transformation into the corresponding virulence measure to allow a comparison to the measured coinfection virulence.

### Calculation of effect sizes

To assess the differences between our different expectations and the observed coinfection virulence, we used Glass’s Δ as a measure of effect size in all 3 analyses and calculated Glass’s Δ and the variance of Glass’s Δ following the calculations found in Marfo and Okyere (2019). We chose to use Glass’s Δ as in our derivation of the mean and the additive expectations we were not able to calculate the corresponding standard deviations. The calculation of Glass’s Δ requires just the standard deviation of 1 comparison group and is thus the most mathematically appropriate effect size measure for these analyses. All models were run initially with ‘study’, ‘parasite one species’, ‘parasite two species’ and ‘host species’ as random effects. In all the measures included, positive values related to higher survival and thus lower virulence. We calculated Glass’s Δ as:

$$\Delta =\frac{\begin{array}{c} survivalexpectationbasedonsingleinfections\\ -survialincoinfection \end{array}}{standarddeviationofsurvialincoinfection}$$

Consequently, positive effect sizes indicate a lower survival and thus higher virulence in the coinfection compared to the expectation based on the single infections. Similarly, negative effect sizes correspond to higher survival and lower virulence in the coinfection.

Variance of Glass’s Δ was calculated as:

$$Variance=\frac{N_{singleinfection}+N_{coinfection}}{N_{singleinfection}*N_{coinfection}}+\frac{\Delta^{2}}{2*(N_{coinfection-1)}}$$

After determining the expectation that best explained virulence levels in coinfection, for the model with this expectation, we carried out moderator variable analyses, testing 10 different moderators: whether the parasites were different strains or different species, whether they shared the same site of infection, whether they shared the same transmission mode, whether the infection dose was double in coinfection, the virulence measure, the level of relatedness, whether 1 of the 2 parasites was transmitted directly, whether one of the parasites could be transmitted from dead hosts and the taxon of each parasite.

### Testing for publication bias

For each of the 3 analyses we created funnel plots to visualize the standard errors in relation to effect size and calculated the fail-safe N (Orwin, 1983).

## Results

### Datasets included

Through our literature research, we found 32 papers containing 68 datasets that fulfilled our inclusion criteria (Figure S1, Table S1). We also found the number of studies investigating coinfections has been increasing over time, beginning in the late 90s and peaking in recent years (Figure S2). This graph is inspired by Griffiths et al. (2011), who addressed the same issue 15 years ago; our data were extracted from the Web of Science independently based on our search terms until 2025.

### Comparison of virulence expectations

**Figure 1.**
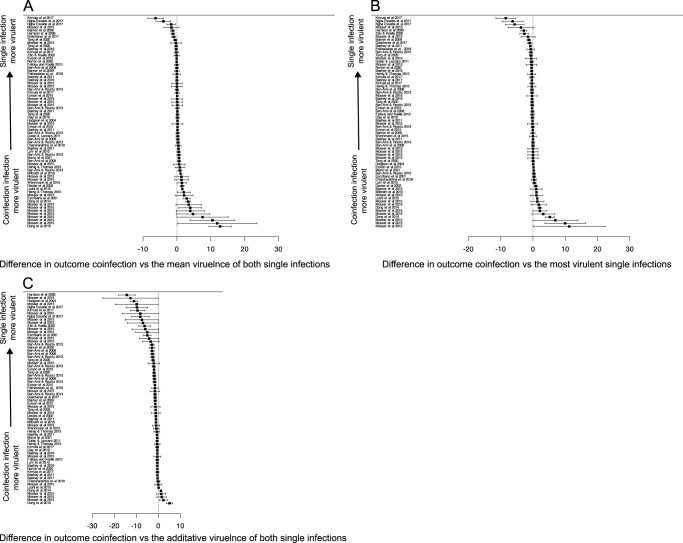
(A) Forest plots showing the effect sizes and 95% confidence intervals for each dataset comparing coinfections to the expectation for mean virulence of both single infections. Positive effect sizes indicate that virulence is higher in coinfection whereas negative values indicate that virulence is lower in coinfection compared to the expected mean virulence of single infections. (B) Forest plots showing the effect sizes and 95% confidence intervals for each dataset comparing coinfections to the most virulent single infection. Positive effect sizes indicate that virulence in higher in coinfection whereas negative values indicate higher virulence of the most virulence single infection. (C) Forest plots showing the effect sizes and 95% confidence intervals for each dataset comparing coinfections to the expectation for additive virulence of both single infections. Positive effect sizes indicate that virulence is higher in coinfection. Negative values indicate that virulence is lower in coinfection compared to the expected additive virulence of single infections. Figure 1 long description.

**Figure 2.**
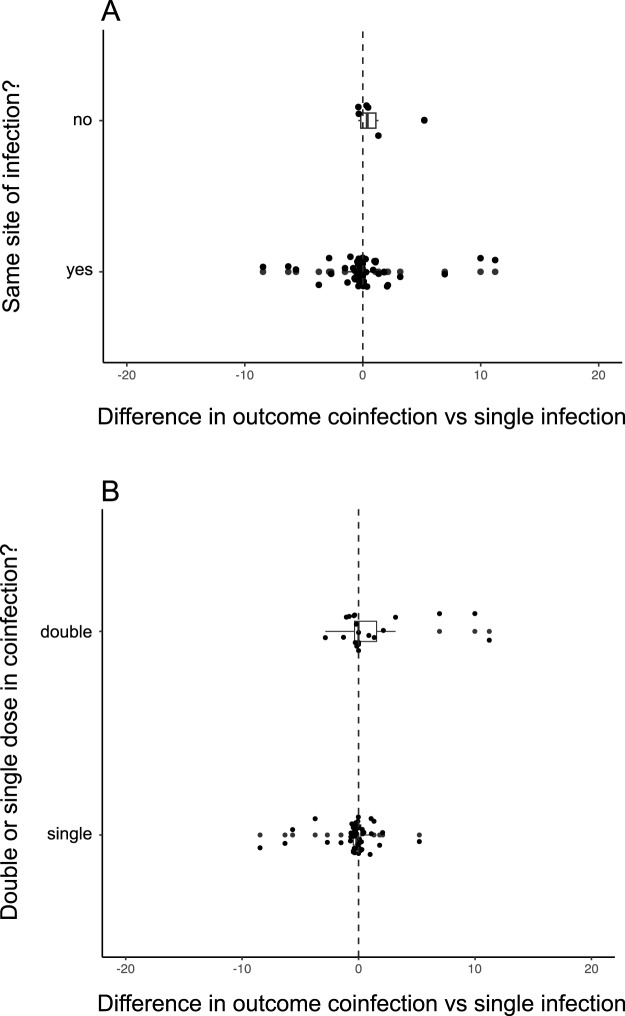
(A) Effect sizes for individual studies grouped by whether the 2 parasites have the same or different sites of infections, using the data from the comparison to the most virulence parasite displayed in Figure 1B. (B) Effect sizes for individual studies grouped by whether the combined dose of both parasite in coinfection was the same or double that of single infections, using the data from the comparison to the most virulence parasite displayed in Figure 1B. Figure 2 long description.

Overall, coinfections (1) were significantly more virulent than the mean of both parasites in single infection (multivariant meta-analysis: Glass’s Δ = 0.87 (95% CI [0.01, 1.74]), p = 0.0484, Figure 1A), (2) did not differ significantly in virulence from most virulent single infection (multivariant meta-analysis: Glass’s Δ = −0.11 (95% CI [−0.63, 0.4]), *p* = 0.6691, Figure 1B) and (3) were significantly less virulent than the additive effect of both parasites in single infection (multivariant meta-analysis: Glass’s Δ = −1.88 (95% CI [−3.03, −0.72]), *p* = 0.0015, Figure 1C). Accordingly, we assumed that among our 3 different expectations, the most virulent single infection best predicts the virulence in a coinfection. Nevertheless, we observed considerable variation in the coinfection virulence, including cases in which coinfection virulence was lower than the expected mean virulence (Figure 1A) and higher than the expected additive virulence (Figure 1C).

### Moderator variable analysis for the comparison of the most virulent parasite to both parasites in coinfection

As our 3 analyses showed that the most virulent parasite seems to be the best predictor of virulence in coinfection, we carried out moderator variable analyses just for the comparison with the most virulent parasite. Of the 10 different moderator variables (Table 2), the only significant moderators were whether or not the 2 species shared an infection site, with those who do not showing higher virulence in coinfection, although the variance for those sharing in infection site was much higher (QM = 6.1284, d.f. = 1, *p* = 0.0133, Figure 2A) (Table 2), although the mean effect size values were positive for both (different infection sites = 1.101074; same infection sites = 0.01869263), and whether the exposure dose was double or single (QM = 4.0438, d.f. = 1, *p* = 0.0443). All infections with parasites of different genotypes of the same species shared an infection site. Nevertheless, the majority of infection sites were shared by parasites of different species. A single dose in coinfection was considered to be half that used in single infection; a double dose was the same amount of parasite used for each single infection in coinfection. For single doses, the median effect size was slightly negative (−0.1839) whereas for double doses it was exactly zero (0.0000), suggesting single infections were slightly more virulent when single doses were administered. There was no difference when double doses were administered.

**Table 2.** Moderator variable analysis for the most virulent single infection Table 2 long description.

Moderator	QM	d.f.	*p* value
**Same infection site?**	**6.1284**	**1**	**0.0133**
Phylogenetic distance	0.8348	1	0.3609
Same transmission mechanism?	0.0014	1	0.9699
**Dose**	**4.0438**	**1**	**0.0443**
Virulence measure	4.58	3	0.205
Transmission of at least 1 parasite via dead host?	0.2682	1	0.6046
Transmission of at least 1 parasite direct?	1.4398	1	0.2302
Different parasite strains of different species?	0.6472	1	0.4211
Taxon parasite A	0.7736	6	0.9928
Taxon parasite B	1.4991	7	0.9823

The bold values represent statistically significant differences

### Publication bias

Funnel plots for all 3 analyses showed indications of publication bias (Figure S3). Fail safe N values were calculated for all 3 analyses, however, and showed that for the result to change a further 1954 effect sizes of null effect would be required for the first analysis with the mean virulence as expectation. As there was no significant difference in the second analysis the fail-safe N was zero. Finally, for the third analysis with the additive expectation, an additional 18 529 effect sizes of null effect would be required. The high fail-safe N values gave us confidence in our results for all 3 analyses, despite the indication of publication bias in the funnel plots.

## Discussion

We found that the most virulent parasite is the best predictor of overall virulence in coinfected hosts. Additive interactions between coinfecting parasites are sometimes assumed, particularly in theory (e.g. Choisy and de Roode, 2010). Although the assumption of additive virulence effects is intuitively logical, our analysis suggests that future models should assume that coinfection is determined instead by the most virulent pathogen in single infection.

Our moderator analysis revealed that parasites sharing a host infection site show a wider range of effects in coinfection. When parasites infect at different sites, their direct interactions are limited, and at the very least delayed, potentially reducing the effects of competition. The mechanisms by which microbes compete within the host are varied, with consequences in evolutionary time and for the outcome of interactions. If parasites must invest heavily in direct competition, they may lack resources to invest in host exploitation. For example, Garbutt et al. (2011) found that when selection lines of *Bacillus thuringiensis* evolve increased antagonism to one another, virulence decreases. Similarly, interference competition reduces virulence of bacteriocin producing bacteria to their caterpillar hosts (Massey et al., 2004).

The studies we included in our analysis enabled us to find broad trends across strains and species. Anthropogenic-induced environmental change is affecting host–parasite interactions, with temperature changes for example shifting host and parasite ranges (Parmesan et al., 1999; Hance et al., 2007; Cahill et al., 2013; Longdon et al., 2015). Changes to the structure of ecological communities increase the likelihood of parasite jumps to novel host species (Hance et al., 2007; Longdon et al., 2015; Hassell et al., 2021). Global change highlights the importance of understanding how the evolutionary history of host–parasite coinfections affects outcomes.

Our study, along with others (Pedersen and Fenton, 2007; Graham, 2008; Griffiths et al., 2014; Rynkiewicz et al., 2015; Betts et al., 2016b; Hassell et al., 2021; Schmitz et al., 2023), highlights the ecological importance and improved understanding that can arise from studying infections by multiple parasites in a host. Specifically, we show across a diverse array of animal–parasite systems that the most virulent parasite drives virulence in coinfection. The treatment strategies for coinfections should focus on the most virulent parasites (as stated in Schmitz et al., 2023).

With coinfections widespread in wildlife (Telfer et al., 2010), and particularly affecting the world’s poorest human populations (Steinmann et al., 2010; Griffiths et al., 2011), and in light of the COVID-19 pandemic (Bai et al., 2021), improving our understanding of the harm caused by coinfections is of vital importance. Here we evaluated the effects of coinfection on mortality-based measures of virulence. Virulence, however, impacts all measures of host fitness and as such there is a need for future meta-analyses to evaluate whether the patterns we see here are also seen in studies measuring virulence via non-mortality measures. This knowledge together with the findings presented here will aid in the effective prediction and control of coinfections outcomes (Read and Taylor, 2001; Vaumourin et al., 2015) and in the development of treatment strategies (Ezenwa and Jolles, 2015; Vaumourin et al., 2015; Ezenwa et al., 2021).

## Supporting information

10.1017/S003118202610170X.sm001 Rafaluk et al. supplementary material 1 Rafaluk et al. supplementary material

10.1017/S003118202610170X.sm002 Rafaluk et al. supplementary material 2 Rafaluk et al. supplementary material

## Supplementary material

The supplementary material for this article can be found at https://doi.org/10.1017/S003118202610170X.

## Table 1 Long description

The table compares mean and additive virulence using different measures of host survival, including proportion survived, percent survived, mean survival time, and LT50. Mean virulence is calculated using the geometric mean of survival rates, while additive virulence uses the product of survival rates. Across all measures, mean virulence results in lower values than additive virulence, suggesting that averaging survival rates results in a less severe impact on host survival. This trend is consistent across all survival measures, highlighting the difference in outcomes when using mean versus additive calculations. The interpretation of these results should consider the assumptions made in transforming survival rates and the potential variability in experimental conditions.

Navigate back to Table 1.

## Table 2 Long description

The table analyzes various moderator variables to determine their impact on the virulence of single infections. Key findings include statistically significant results for 'Same infection site?' and 'Dose', with p values of 0.0133 and 0.0443, suggesting these factors may significantly affect virulence. Other variables, such as 'Phylogenetic distance' and 'Same transmission mechanism?', did not show significant differences, with p values of 0.3609 and 0.9699, respectively. The table also examines the influence of different parasite strains, taxon of parasites, and transmission methods, but these did not yield significant results. The analysis highlights the importance of infection site and dose in understanding virulence, while other factors may require further investigation to determine their roles.

Navigate back to Table 2.

## Figure 1 Long description

The image A shows a forest plot comparing coinfection virulence to the mean virulence of both single infections. The x-axis is labeled 'Difference in outcome coinfection vs the mean virulence of both single infections', with values ranging from negative 10 to 30. The y-axis lists various datasets. Positive effect sizes indicate higher virulence in coinfection, while negative values indicate lower virulence compared to the mean of single infections. The image B shows a forest plot comparing coinfection virulence to the most virulent single infection. The x-axis is labeled 'Difference in outcome coinfection vs the most virulent single infections', with values ranging from negative 20 to 30. The y-axis lists various datasets. Positive effect sizes indicate higher virulence in coinfection, while negative values indicate higher virulence of the most virulent single infection. The image C shows a forest plot comparing coinfection virulence to the additive virulence of both single infections. The x-axis is labeled 'Difference in outcome coinfection vs the additive virulence of both single infections', with values ranging from negative 30 to 10. The y-axis lists various datasets. Positive effect sizes indicate higher virulence in coinfection, while negative values indicate lower virulence compared to the expected additive virulence of single infections

Navigate back to Figure 1.

## Figure 2 Long description

The image contains two scatter plots. The first plot, labeled A, compares the difference in outcome between coinfection and single infection based on whether the infection site is the same or different. The y-axis is labeled 'Same site of infection?' with 'no' and 'yes' as categories. The x-axis is labeled 'Difference in outcome coinfection vs single infection' with values ranging from negative 20 to positive 20. Data points are scattered around the zero line, with a cluster near zero for both categories. The second plot, labeled B, examines the difference in outcome based on whether the dose in coinfection is double or single. The y-axis is labeled 'Double or single dose in coinfection?' with 'double' and 'single' as categories. The x-axis is the same as in plot A. Data points are similarly scattered around the zero line, with a concentration near zero for both dose categories. Both plots feature a vertical dashed line at zero, indicating no difference in outcome between coinfection and single infection.

Navigate back to Figure 2.
