# Supplementary material for: The most virulent parasite determines virulence in coinfection: a meta-analysis
Source: Parasitology. 2026 Feb 11;153(4):540–8. doi: 10.1017/S003118202610170X (PMC13244233; doi:10.1017/S003118202610170X)
Supplement: Rafaluk et al. supplementary material 1 — Rafaluk et al. supplementary material [file S003118202610170Xsup001.pdf]

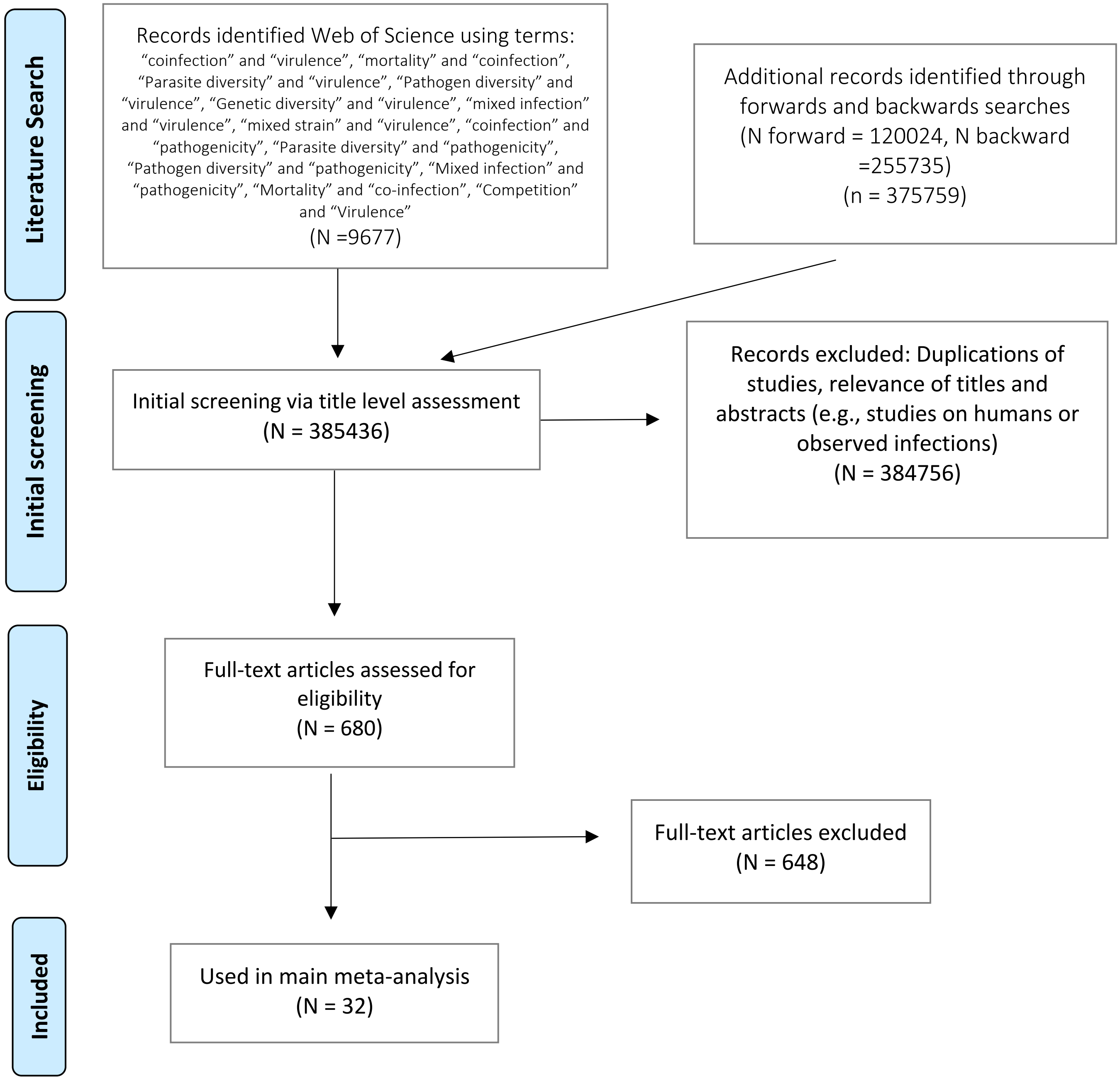

Figure S1: PRISMA flow chart detailing data collection (Moher *et al.*, 2009)

From: Moher D, Liberati A, Tetzlaff J, Altman DG, The PRISMA Group (2009). *P*referred *R*eporting *I*tems for *S*ystematic Reviews and *M*eta-*A*nalyses: The PRISMA Statement. PLoS Med 6(7): e1000097. doi:10.1371/journal.pmed1000097

For more information, visit www.prisma-statement.org.

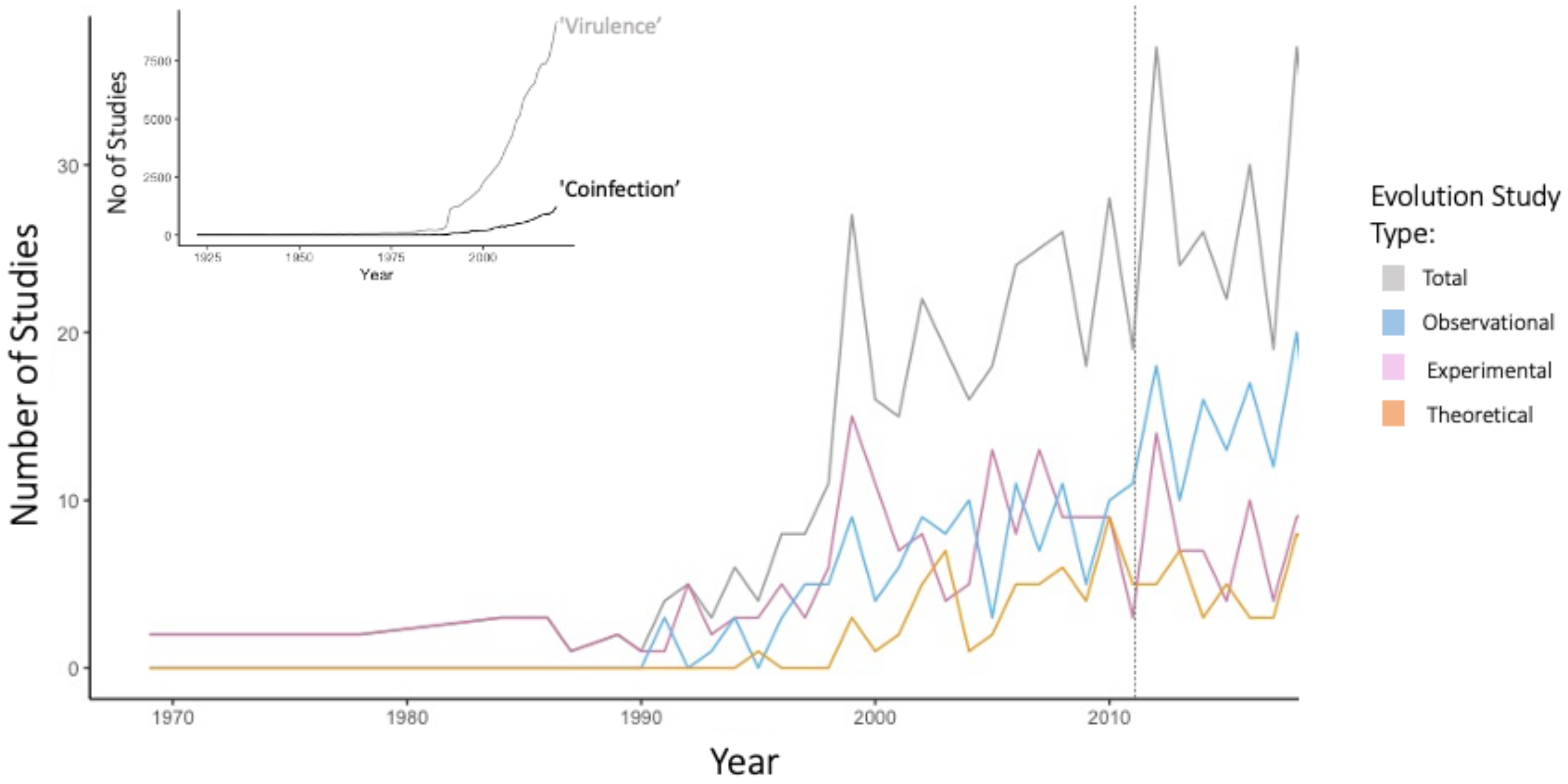

Figure S2: The number of coinfection studies over time. The large graph represents those within the field of evolutionary ecology and the smaller graph shows the total number. The dotted line represents the end of Griffiths et al 2011 sampling. Data was collected from Web of Science. The figure provided as an inset in the top left provides a comparison between the total number of published studies focused on coinfection and the total number focused on virulence during the same time period.

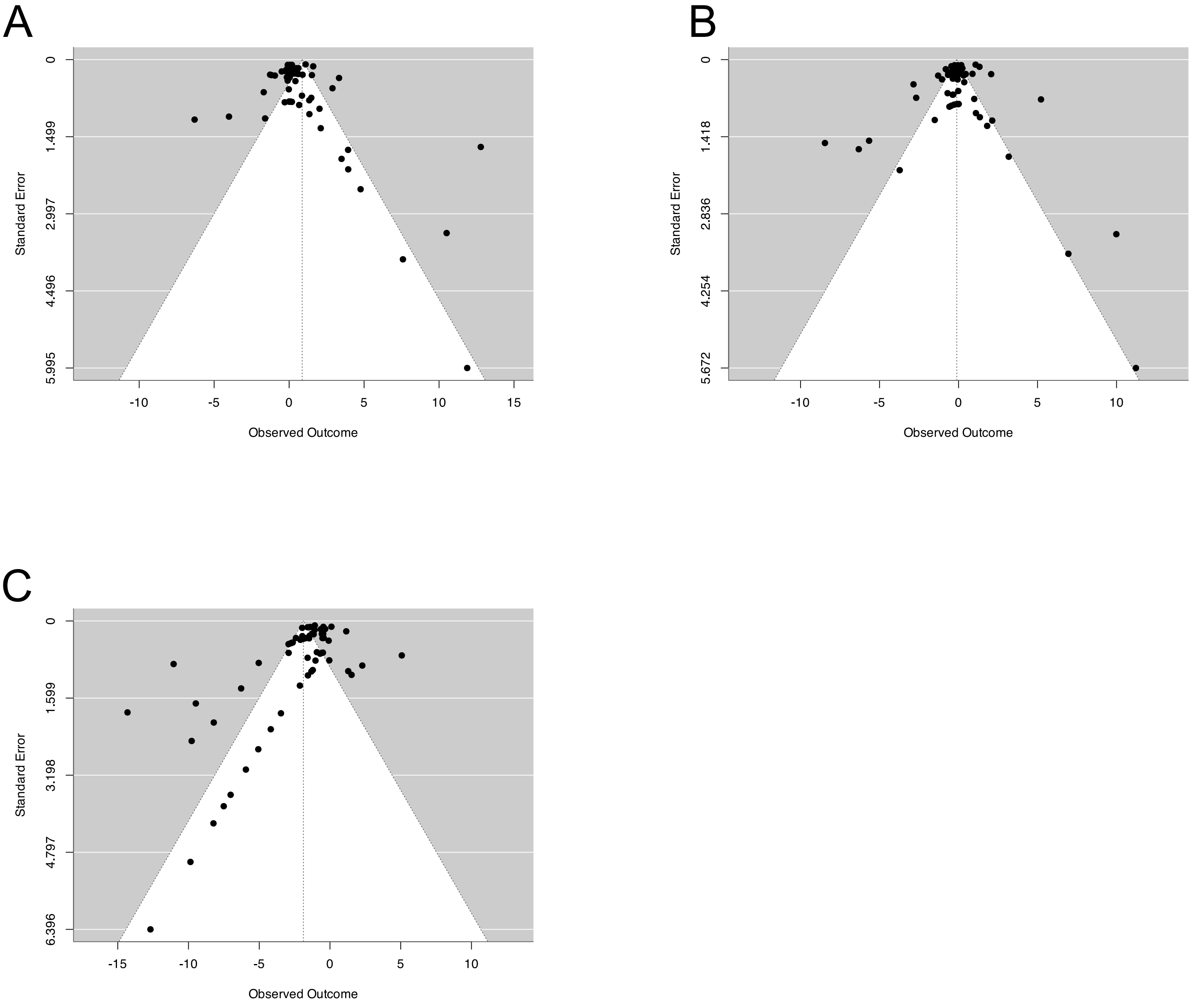

Figure S3. Funnel plots for each of the meta-analyses. A) Comparing coinfections to the mean virulence of both single infections; B) comparing coinfections to the most virulent parasite in single infection and C) comparing coinfections to the additive effects of both parasites in single infection
